# Supplementary material for: Symptom improvement and predictors associated with improvement after 6 weeks of alpha-blocker therapy: An exploratory, single-arm, open-label cohort study
Source: PLoS One. 2019 Jul 25;14(7):e0220417. doi: 10.1371/journal.pone.0220417 (PMC6657904; doi:10.1371/journal.pone.0220417)
Supplement: S1 Table — (DOCX) [file pone.0220417.s002.docx]

| Medication classified by ATC-code | Percentage of patients |
| --- | --- |
| C10 [Lipid Modifying Agents](https://www.whocc.no/atc_ddd_index/?code=C10) | 34% |
| B01 [Antithrombotic Agents](https://www.whocc.no/atc_ddd_index/?code=B01) | 28% |
| A02 [Drugs For Acid Related Disorders](https://www.whocc.no/atc_ddd_index/?code=A02) | 27% |
| C09 [Agents Acting On The Renin-Angiotensin System](https://www.whocc.no/atc_ddd_index/?code=C09) | 24% |
| C03 [Diuretics](https://www.whocc.no/atc_ddd_index/?code=C03) | 14% |
| C07 [Beta Blocking Agents](https://www.whocc.no/atc_ddd_index/?code=C07) | 16% |
| A10 [Drugs Used In Diabetes](https://www.whocc.no/atc_ddd_index/?code=A10) | 13% |
| C08 [Calcium Channel Blockers](https://www.whocc.no/atc_ddd_index/?code=C08) | 13% |
| R03 [Drugs For Obstructive Airway Diseases](https://www.whocc.no/atc_ddd_index/?code=R03) | 10% |
| G04 [Urologicals](https://www.whocc.no/atc_ddd_index/?code=G04) | 9% |
| A06 [Drugs For Constipation](https://www.whocc.no/atc_ddd_index/?code=A06) | 8% |
| R01 [Nasal Preparations](https://www.whocc.no/atc_ddd_index/?code=R01) | 6% |
| C01 [Cardiac Therapy](https://www.whocc.no/atc_ddd_index/?code=C01) | 5% |
