# Supplementary material for: Symptom improvement and predictors associated with improvement after 6 weeks of alpha-blocker therapy: An exploratory, single-arm, open-label cohort study
Source: PLoS One. 2019 Jul 25;14(7):e0220417. doi: 10.1371/journal.pone.0220417 (PMC6657904; doi:10.1371/journal.pone.0220417)
Supplement: S2 Table — Predictors of clear improvement in PGI-I. AUC = Area Under the Curve; NI = Not included. (DOCX) [file pone.0220417.s003.docx]

|  | Univariable analysis | | Multivariable analysis | |
| --- | --- | --- | --- | --- |
|  | OR | 95% CI | OR | 95% CI |
| Age (ref = <60) |  |  |  |  |
| *60-70* | 1.52 | (0.56;41.4) | NI |  |
| *>70* | 1.00 | (0.35;2.85) | NI |  |
| Duration of complaints (ref = <6 months) |  |  |  |  |
| *6-24 months* | 0.27 | (0.06;1.14) | 0.23 | (0.05;1.12) |
| *> 24 months* | **0.38** | (**0.16;0.94**) | **0.36** | (**0.13;0.98**) |
| IPSS sum score | 1.01 | (0.95;1.07) | NI |  |
| IPSS storage baseline | 0.94 | (0.88;1.13) | NI |  |
| IPSS voiding baseline | 1.02 | (0.94;1.11) | NI |  |
| OABq-sf baseline | 1.01 | (0.99;1.03) | NI |  |
| Still using alpha-blockers at 6 weeks (ref = no) | **6.52** | (**1.80;23.60**) | **8.03** | (**1.96;32.95**) |
| Comorbidity (ref = no) | 1.24 | (0.50;3.07) | NI |  |
| Prostate abnormal (ref = no) |  |  |  |  |
| *Increased size* | 1.12 | (0.43;2.91) | NI |  |
| *Decreased size* | 2.00 | (0.12;34.82) | NI |  |
| *Not examined* | 1.54 | (0.52;4.52) | NI |  |
| Examination of pelvic floor (ref = hypertonic) |  |  |  |  |
| *Not hypertonic* | 2.81 | (0.30;28.98) | NI |  |
| *Not determined* | 3.21 | (0.32;32.21) | NI |  |
| *Not examined* | 3.75 | (0.37;37.95) | NI |  |
|  |  |  |  |  |
| Number of co-medication (ref = 0-1) |  |  |  |  |
| *2-5* | 1.24 | (0.49;3.15) | 0.78 | (0.28;2.22) |
| *6 or more* | **3.75** | (**1.19;11.81**) | 3.24 | (0.92;11.41) |
| Co-medication with an effect on LUTS (ref = no) | 0.97 | (0.26;3.56) | NI |  |
|  |  |  | Nagelkerk R^2^ = 27%  Hosmer-Lemeshow test = 0.90  AUC = 0.76 | |
