# Supplementary material for: Symptom improvement and predictors associated with improvement after 6 weeks of alpha-blocker therapy: An exploratory, single-arm, open-label cohort study
Source: PLoS One. 2019 Jul 25;14(7):e0220417. doi: 10.1371/journal.pone.0220417 (PMC6657904; doi:10.1371/journal.pone.0220417)
Supplement: S3 Table — Predictors of improvement in IPSS. NI = Not included; *NI due to multicollinearity. (DOCX) [file pone.0220417.s004.docx]

|  | Univariable analysis | | Multivariable analysis | |
| --- | --- | --- | --- | --- |
|  | IPSS change | 95% CI | IPSS change | 95% CI |
| Constant |  |  |  |  |
| Age (ref = <60) |  |  |  |  |
| *60-70* | 1.38 | (-2.07;4.85) | NI |  |
| *>70* | 0.06 | (-3.49;3.61) | NI |  |
| Duration of complaints (ref = <6 months) |  |  |  |  |
| *6-24 months* | 0.76 | (-3.85;5.37) | NI |  |
| *> 24 months* | 0.03 | (-3.08;3.13) | NI |  |
| IPSS sum score | **-0.61** | (**-0.78;-0.44**) | **-0.63** | (**-0.85;-0.42**) |
| IPSS storage baseline | **-0.92** | (**-1.32;-0.53**) | NI* |  |
| IPSS voiding baseline | **-0.74** | (**-0.98;-0.50**) | NI* |  |
| OABq-sf baseline | **-0.09** | (**-0.16;-0.02**) | 0.04 | (-0.03;0.11) |
| Still using alpha-blockers at 6 weeks (ref = no) | -0.67 | (-3.87;2.54) | NI |  |
| Comorbidity (ref = no) | -1.54 | (-4.70;1.61) | NI |  |
| Prostate abnormal (ref = no) |  |  |  |  |
| *Increased size* | -0.76 | (-3.99;2.47) | NI |  |
| *Decreased size* | 8.47 | (-1.68;18.62) | NI |  |
| *Not examined* | -0.88 | (-4.61;2.85) | NI |  |
| Examination of pelvic floor (ref = hypertonic) |  |  |  |  |
| *Not hypertonic* | 1.77 | (-4.36;7.91) | NI |  |
| *Not determined* | 1.42 | (-5.09;7.93) | NI |  |
| *Not examined* | -1.52 | (-5.05;8.10) | NI |  |
|  |  |  |  |  |
| Number of co-medications (ref = 0-1) |  |  |  |  |
| *2-5* | -0.82 | (-3.80;2.15) | 0.42 | (-2.14;2.97) |
| *6 or more* | **-6.11** | (**-9.87;-2.36**) | -1.97 | (-5.42;1.47) |
| Co-medication with an effect on LUTS (ref = no) | -0.23 | (-4.72;4.27) |  |  |
|  |  |  | Adjusted R^2^ = 34.0% | |
