# Supplementary material for: Symptom improvement and predictors associated with improvement after 6 weeks of alpha-blocker therapy: An exploratory, single-arm, open-label cohort study
Source: PLoS One. 2019 Jul 25;14(7):e0220417. doi: 10.1371/journal.pone.0220417 (PMC6657904; doi:10.1371/journal.pone.0220417)
Supplement: S4 Table — Predictors of improvement in OABq-SF. NI = Not included; *NI due to multicollinearity. (DOCX) [file pone.0220417.s005.docx]

|  | Univariable analysis | | Multivariable analysis | |
| --- | --- | --- | --- | --- |
|  | OABq-SF change | 95% CI | OABq-SF change | 95% CI |
| Constant |  |  |  |  |
| Age (ref = <60) |  |  |  |  |
| *60-70* | 2.71 | (-5.94;11.35) | NI |  |
| *>70* | 3.44 | (-5.43;12.32) | NI |  |
| Duration of complaints (ref = <6 months) |  |  |  |  |
| *6-24 months* | 3.81 | (-7.66;15.27) | NI |  |
| *> 24 months* | 3.13 | (-4.60;10.85) | NI |  |
| IPSS sum score | **-0.85** | (**-1.34;-0.36**) | -0.06 | (-0.57;0.45) |
| IPSS storage baseline | **-1.56** | (**-2.60;-0.52**) | NI* |  |
| IPSS voiding baseline | **-0.91** | (**-1.59;-0.23**) | NI* |  |
| OABq-sf baseline | **-0.52** | (**-0.67;-0.37**) | **-0.50** | (**-0.68;-0.32**) |
| Still using alpha-blockers at 6 weeks (ref = no) | -6.08 | (-13.99;1.83) | -3.29 | (-9.94;3.35) |
| Comorbidity (ref = no) | 1.20 | (-6.70;9.11) | NI |  |
| Prostate abnormal (ref = no) |  |  |  |  |
| *Increased size* | 2.81 | (-5.25;10.87) | NI |  |
| *Decreased size* | 22.19 | (-3.13;47.52) | NI |  |
| *Not examined* | -0.33 | (-9.63;8.98) | NI |  |
| Examination of pelvic floor (ref = hypertonic) |  |  |  |  |
| *Not hypertonic* | 2.58 | (-12.57;17.75) | NI |  |
| *Not determined* | 8.58 | (-7.50;24.66) | NI |  |
| *Not examined* | 3.19 | (-13.05;19.43) | NI |  |
|  |  |  |  |  |
| Number of co-medications (ref = 0-1) |  |  |  |  |
| *2-5* | -4.06 | (-11.79;3.68) | NI |  |
| *6 or more* | -6.48 | (-16.25;3.30) | NI |  |
| Co-medication with an effect on LUTS (ref = no) | -5.92 | (-17.08;5.24) | NI |  |
|  |  |  | Adjusted R^2^ = 31.6% | |
