# Supplementary material for: Symptom improvement and predictors associated with improvement after 6 weeks of alpha-blocker therapy: An exploratory, single-arm, open-label cohort study
Source: PLoS One. 2019 Jul 25;14(7):e0220417. doi: 10.1371/journal.pone.0220417 (PMC6657904; doi:10.1371/journal.pone.0220417)
Supplement: Attachment — Submitted filename: Response to Reviewers.docx [file pone.0220417.s006.docx]

Reply to editor comments:

When submitting your revision, we need you to address these additional requirements.

1. Please ensure that your manuscript meets PLOS ONE's style requirements, including those for file naming.

We noted some errors in the manuscript style (capitals in title) and corrected those (Page 3, line 57 and page 9, line 150)

2. Please include additional information regarding the survey or questionnaire used in the study and ensure that you have provided sufficient details that others could replicate the analyses. For instance, if you developed a questionnaire as part of this study and it is not under a copyright more restrictive than CC-BY, please include a copy, in both the original language and English, as Supporting Information.  Moreover, please define the follow-up period for this study.

For this study we used the Dutch versions of the IPSS and the OAB-q that have been referenced in the manuscript. We did not develop a questionnaire but only asked for some demographic and disease specific information, in addition to aforementioned questionnaires. These questions have been described in the manuscript (page 4, lines 73-74 and 83-85). As the questions were in Dutch, we doubt if we needed to make the complete questionnaire available.

3. We note that you have indicated that data from this study are available upon request. PLOS only allows data to be available upon request if there are legal or ethical restrictions on sharing data publicly. If there are no restrictions, please upload the minimal anonymized data set necessary to replicate your study findings as either Supporting Information files or to a stable, public repository.

There are no legal or ethical restrictions on sharing data. We have now added a minimal anonymized dataset as a supporting Information file.

Reply to reviewer comments:

1) Need to specify the medications that were consumed by participants but deemed to not have an effect on LUTS by the authors. How many such medications were used by patients.

We made a frequency table of the other medications that were used. We presented the medications that were used by ≥5% percent of the participants in a supplementary file (S1 Table). And we referred to this table in the results section:

Page 6, line 135: *An overview of the medication that was used by the patients is given in supporting file S1 Table.*

2) Considering the 3.89 OR for PGI improvement with 6 medications, it is likely that the assumption of medications not having an effect on LUTS is untrue or the medications are affecting urine production.

We agree that it might be possible that other medications could influence LUTS or urine production, but we do not feel that this is very likely. First of all and most important, the other medication was initiated in the period preceding baseline assessment and had not changed during the observation period. Therefore it is not likely that symptoms improved during follow-up because of that medication. Secondly, we controlled for medication that is known to have an effect on urinary symptoms and we did not find a significant association between this variable and the outcomes. Because of this we feel it is unlikely that other medication affected outcomes. We therefore sought an explanation for the relation between the use of multiple types of medication and improvement of symptoms elsewhere, and found several studies that have shown a link between the use of multiple types of medication and adherence. For transparency we have provided a list of the most used types of medication (S1 Table). We added the following to the Discussion:

Page 17, lines 221-226: *Another explanation is that other types of medication lead to improvement of LUTS, however this is less likely. First of all, other medication was initiated before baseline, and remained unchanged during the observation period. Secondly, in our analyses we looked at known specific drugs with the potential to affect LUTS and found no relation with change in symptoms, although we cannot rule out that there are other types of drugs that have an influence on LUTS.*

3)Table 3 and 4 have negative values as predictors, does the negative sign imply that the variable worsens the LUTS

A lower score on the IPSS means less symptoms and therefore a negative sign in the regression coefficients indicates an improvement of symptoms. To emphasize this we have added this information to the Materials en Methods section and to the footnotes of the two tables.

Page 6, line 127: *Because a higher IPSS score indicates more symptoms, regression coefficients of improvement will have a negative sign.*

Table footnote: *Negative regression coefficients indicate improvement of symptoms.*

4) scores on (i.e., the IPSS and OAB-q SF) are subjective and not objective measures of improvement

We agree that both the IPSS and OAB-q SF are not objective measures of symptoms. The point we tried to make is that both questionnaires are more objective than the PGI-I because they ask for measurable events such as the frequency of urination, whereas the PGI-I asks for the more qualitative impression of improvement. We now replaced the term ‘objective’ with ‘quantitative’ to make this more clear.

Page 16, line 213: *However, no such relationship existed between current alpha-blocker use and the more quantitative measures of improvement (i.e., the IPSS and OAB-q SF), suggesting that any observed improvements in symptoms could not be attributed to changes in either the frequency of urination or episodes of incontinence.*
